# Primary pancreatic-type acinar cell carcinoma of the jejunum with tumor thrombus extending into the mesenteric venous system: a case report and literature review

**DOI:** 10.1186/s12893-017-0273-3

**Published:** 2017-06-29

**Authors:** Kosei Takagi, Takahito Yagi, Takehiro Tanaka, Yuzo Umeda, Ryuichi Yoshida, Daisuke Nobuoka, Takashi Kuise, Toshiyoshi Fujiwara

**Affiliations:** 1 Department of Gastroenterological Surgery, Okayama University Graduate School of Medicine, Dentistry and Pharmaceutical Sciences, 2-5-1 Shikata-cho, Kita-ku, Okayama, 700-8558 Japan; 2 Department of Diagnostic Pathology, Okayama University Hospital, 2-5-1 Shikata-cho, Kita-ku, Okayama, 700-8558 Japan

**Keywords:** Acinar cell carcinoma, Jejunum, Ectopic pancreas, Tumor thrombus

## Abstract

**Background:**

Although ectopic pancreatic tissue is common in the upper gastrointestinal tract, the incidence of ectopic pancreatic tissue in the jejunum is low, and malignant transformation in ectopic pancreatic tissue is rare. Furthermore, pancreatic-type acinar cell carcinoma (ACC) developing in the jejunum and ACC accompanied by tumor thrombus are extremely rare.

**Case presentation:**

A 78-year-old-woman presented with melena. Abdominal computed tomography images and endoscopic examination revealed a submucosal jejunal mass with tumor thrombus extending into a jejunal vein. The patient underwent a curative resection combined with a partial jejunectomy and partial pancreatectomy. Histopathological examination of the resected tissue showed tumor cells with a homogeneous acinar architecture identical to pancreatic-type ACC and tumor thrombus. Postoperatively, she was followed for 10 months and had no recurrence.

**Conclusion:**

We present an extremely rare case of pancreatic-type ACC in the jejunum with extensive tumor thrombus invading into the mesenteric venous system. This type of cancer has not been reported previously but should be considered in the differential diagnosis of a jejunal mass.

## Background

Ectopic pancreatic tissue is most frequently found in the upper gastrointestinal tract, and malignant transformation of ectopic pancreatic tissue is rare. In the English literature, approximately 30 cases of adenocarcinoma arising from ectopic pancreatic tissue have been reported, and most occurred in the upper gastrointestinal tract [1, 2]. Only seven cases of adenocarcinoma arising from ectopic pancreatic tissue in the jejunum have been reported [3].

Acinar cell carcinoma (ACC) is a rare malignant tumor that accounts for 1–2% of all exocrine pancreatic neoplasms and usually arises in the pancreatic parenchyma [4]. ACC derived from ectopic pancreatic tissue is very rare, with only 12 previous reports [1, 2, 5–14]. Among these reports, only one described ACC in jejunal pancreatic heterotopia [14]. Furthermore, ACC accompanied by tumor thrombus is very rare. In the English literature, there have been only two reports of ACC with portal vein tumor thrombus [15, 16].

We herein report an extremely rare case of pancreatic-type ACC presenting as a submucosal jejunal tumor accompanied by tumor thrombus and review the previously reported cases of heterotopic ACC.

## Case presentation

A 78-year-old woman was admitted to another hospital complaining of melena and transferred to our facility for further examination. Her physical examination was unremarkable. The laboratory values were as follows: white blood count, 4220 cells/μL; hemoglobin level, 12.4 g/dL; platelet count, 16.4 × 10^4^ cells/μL; aspartate transaminase, 25 IU/L; alanine aminotransferase, 16 IU/L; total bilirubin, 0.8 mg/dL; albumin, 3.6 g/dL; and creatinine, 0.48 mg/dL. Tumor markers (carcinoembryonic antigen, carbohydrate antigen 19–9, s-pancreas-1 antigen, and Duke pancreatic monoclonal antigen type 2) were within normal limits.

**Fig. 1:**
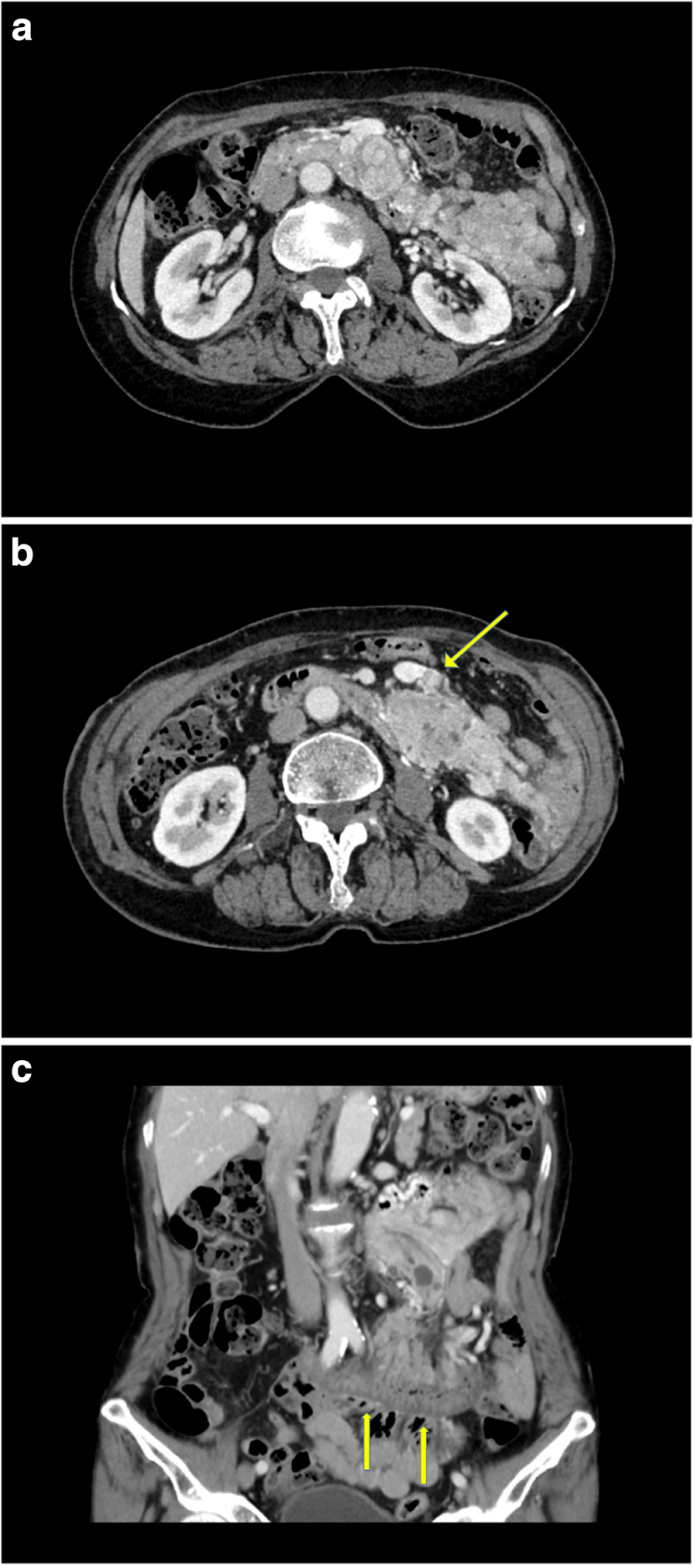
Preoperative computed tomography scans: **a**. An 8.5 × 4.0 cm exophytic submucosal tumor located in the jejunum. The posterior aspect of the superior mesenteric vein is compressed by the tumor, and the tumor extends to the uncinate process of the pancreas; **b**. The tumor thrombus invades the first jejunal vein (*arrow*); **c.** The jejunal mesentery shows significant thickening (*arrow*)

Abdominal computed tomography scans revealed an 8.5 × 4.0 cm exophytic submucosal tumor in the jejunum (Fig. 1a). The tumor extended to the uncinate process of the pancreas and compressed the posterior aspect of the superior mesenteric vein. The first jejunal vein was thrombosed, and we suspected a tumor thrombus. Tumor thrombus was not identified in the superior mesenteric vein (Fig. 1b). We also observed significant thickening of the jejunal mesentery, but no distant metastasis was found (Fig. 1c).

**Fig. 2:**
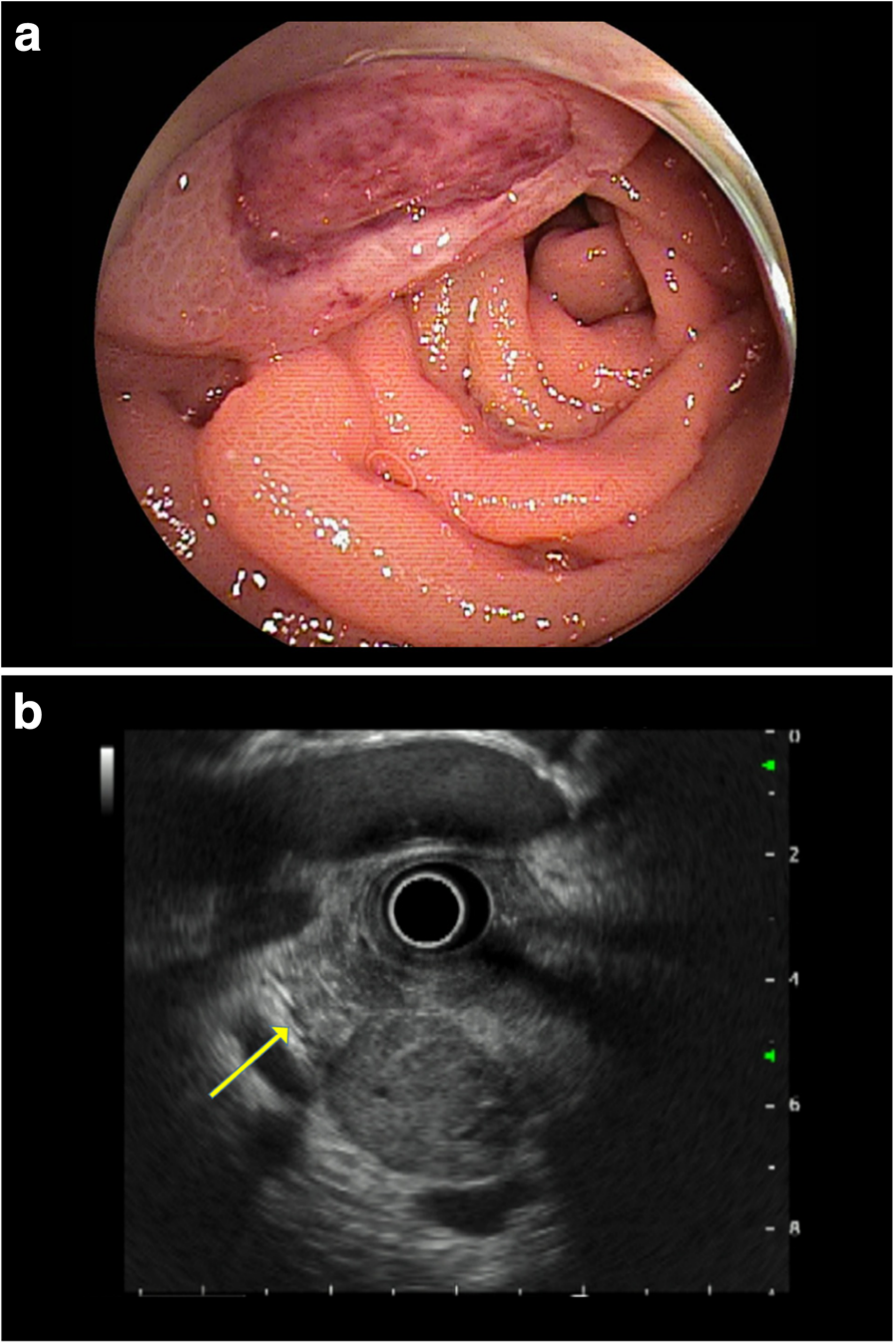
Endoscopic examination: **a**. Double-balloon enteroscopy reveals a centrally ulcerated submucosal jejunal lesion measuring approximately 3 cm; **b**. Endoscopic ultrasonography shows a submucosal tumor touching the uncinate process of the pancreas, suggesting the possibility of pancreatic infiltration (*arrow*, pancreas)

Double-balloon enteroscopy showed an approximately 3 cm submucosal tumor with central ulceration in the jejunum (Fig. 2a). Endoscopic biopsy revealed a poorly differentiated adenocarcinoma. Endoscopic ultrasonography showed a submucosal jejunal tumor which touched the uncinate process of the pancreas and suggested possible pancreatic infiltration (Fig. 2b).

Our preoperative diagnosis was a jejunal adenocarcinoma. Although there was tumor thrombus in the mesenteric venous system and the possibility of pancreatic invasion, we considered the tumor resectable.

At surgery, there was neither peritoneal dissemination nor liver metastasis. The tumor was located mainly in the mesentery of the upper jejunum. No invasion of the superior mesenteric artery or vein was found. We did not observe invasion of the pancreatic uncinate process grossly, but we resected it. Accordingly, the patient underwent a curative resection combined with a partial jejunectomy (approximately 50 cm of the jejunum) and a partial pancreatectomy (uncinate process).

**Fig. 3:**
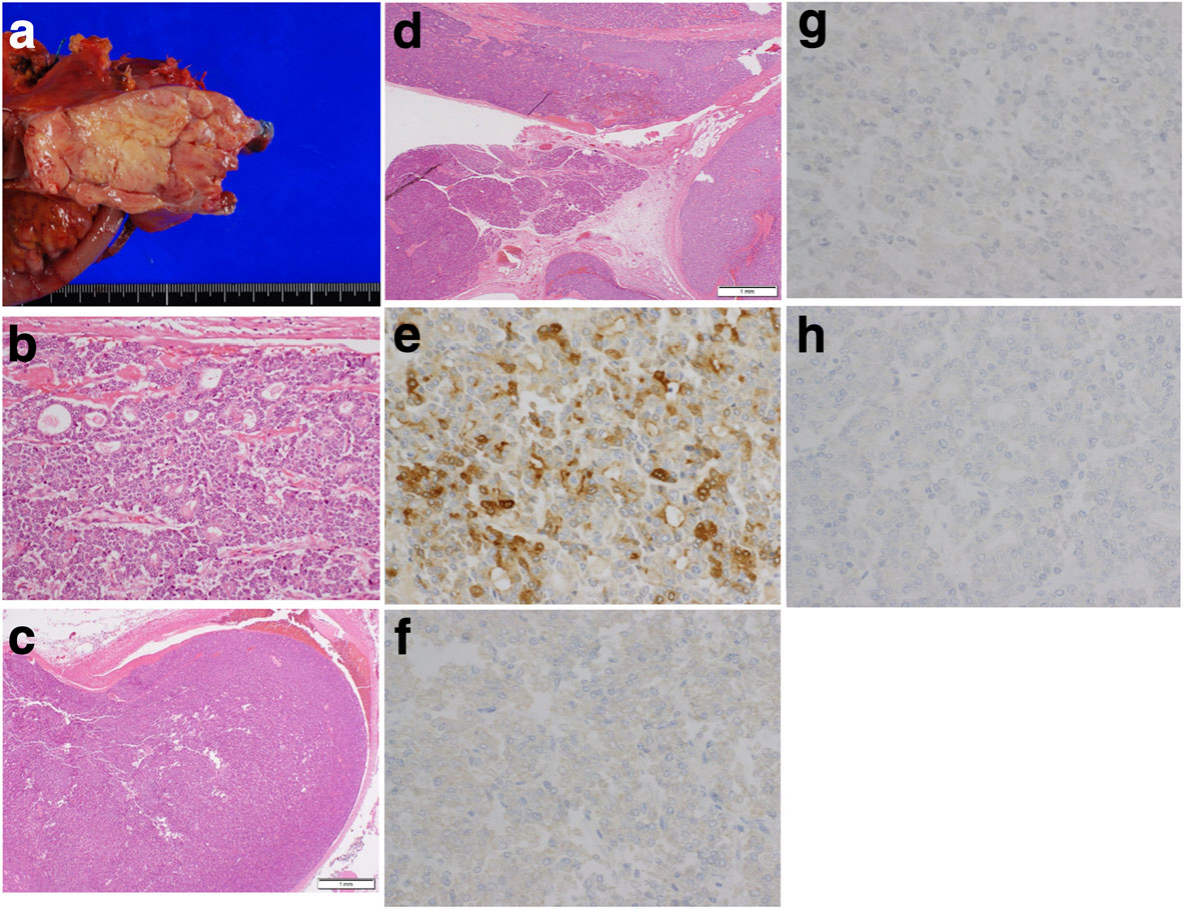
Pathological examination of the jejunal tumor: **a**. Gross examination shows an 8.5 cm, soft, circumscribed, yellowish-white, submucosal mass in the jejunum; **b**. Microscopic examination reveals acinar and solid architectural patterns; **c**. Marked vascular invasion, including tumor thrombus, extends into the first jejunal vein; **d**. No invasion to the pancreatic parenchyma is identified; Immunohistochemically, the tumor cells are positive for trypsin (**e**) and negative for chromogranin A (**f**), synaptophysin (**g**), and CD56 (**h**)

The gross examination revealed an 8.5 cm, soft, circumscribed, yellowish-white, submucosal mass in the jejunum (Fig. 3a). Histologically, the tumor showed an acinar and solid growth pattern (Fig. 3b). Marked vascular invasion was observed, and the tumor thrombus extended into the first jejunal vein (Fig. 3c). The tumor was confined to the jejunal wall without pancreatic parenchymal invasion (Fig. 3d). Immunohistochemically, the tumor cells were positive for trypsin and negative for chromogranin A, synaptophysin, and CD56 (Figs. 3e, f, g, and h). The resected margins were free of tumor cells. Although residual ectopic pancreas was not identified, the tumor was diagnosed as pancreatic-type ACC of the jejunum based on the architectural patterns of ACC and immunohistochemical findings.

The postoperative course was uneventful. The patient received adjuvant chemotherapy with S-1 (TS-1; Taiho Pharmaceutical, Tokyo, Japan) after the curative resection. She was alive and without recurrence at her last follow-up visit 10 months postoperatively.

## Discussion and conclusions

To the best of our knowledge, this is the first report of pancreatic-type ACC in the jejunum accompanied by tumor thrombus in the mesenteric venous system. This report highlights the clinicopathological findings of an extremely rare case and reviews the features of previously reported cases of heterotopic ACC.

There have been only 13 cases, including our case, reporting heterotopic ACC in a digestive organ (Table 1). The cases included seven women and six men with a mean age of 61.8 years. Heterotopic ACC was most frequently found in the upper gastrointestinal tract. The average tumor size was relatively large (4.5 cm). Interestingly, no case was preoperatively diagnosed as ACC. Indeed, five cases were diagnosed as poorly differentiated adenocarcinoma by biopsy, and other tumor types were suspected in four cases. ACCs usually develop submucosally, so preoperative diagnosis by histological examination might be difficult. Furthermore, the histological examination of the resected specimen revealed ectopic pancreatic tissue in only two cases.

**Table 1:** Demographic and clinicopathological features in reported cases of acinar cell carcinoma arising from heterotopic pancreas

Reports	Age^a^ /Sex	Site	Size (cm)	Preoperative diagnosis	Metastasis	Tumor characteristics	Treatment	Adjuvant chemo	EPT	Outcome
Sun et al. 2004 [5]	86/F	Stomach	5.0	PDA	None	Exophytic, ulcerated	Partial gastrectomy	NM	A	NM
Mizuno et al. 2007 [6]	73/M	Stomach	7.6	GIST/L	LN	Submucosal	PD	NM	A	11 months alive, liver metastasis at 7 months
Ambrosini-Spaltro et al. 2009 [7]	52/M	Stomach	4.0	PDA	None	Ulcerated	Subtotal gastrectomy	NM	P	NM
Coyne et al. 2012 [8]	77/F	Stomach	4.5	PDA	None	Ulcerated, submucosal	Partial gastrectomy	NM	A	NM
Yonenaga et al. 2016 [2]	63/M	Stomach	6.5	PDA	Liver	Borrmann type-2 lesion	Chemo	None	A	5 month died, sepsis
Kim et al. 2017 [9]	54/M	Stomach	2.7	GIST/L	None	Submucosal	Wedge resection	None	A	33 months alive, NR
Bookman 1932 [10]	28/F	Duodenum	NM	Benign^b^	None	NM	Partial resection	NM	A	NM
Jahromi et al. 2013 [1]	58/M	Duodenum	2.7	NM	None	Submucosal	Partial resection	Cap + Oxal	A	18 months alive, NR
Kawakami et al. 2007 [11]	65/F	AoV	1.2	NM	None	Exophytic	PD	NM	A	19 months alive, NR
Hervieu et al. 2008 [12]	35/F	Liver	4.0	HCC	None	Well-limited	Hepatectomy	None	A	6 years alive, NR
Chiaravalli et al. 2009 [13]	65/F	Colon	4.0	NM	LN	Ulcerated	Partial resection	NM	A	24 months died, bone metastasis at 18 months
Makhlouf et al. 1999 [14]	71/M	Jejunum	3.5	NM	None	Ulcerated	Partial resection	NM	P	1 year alive, liver metastasis at 1 year
Our case	76/F	Jejunum	8.5	PDA	None	Exophytic, ulcerated, submucosal, mass with tumor thrombus	Partial resection with partial pancreatectomy	S-1	A	10 months alive, NR

*F* female, *M* male, *P* present, *A* absent, *PDA* poorly differentiated adenocarcinoma, *NM* not mentioned, *GIST/L* gastrointestinal stromal tumor or lymphoma, *HCC* hepatocellular carcinoma, *AoV* ampulla of Vater, *Cap + Oxal* Capecitabine + Oxaliplatin, *PD* pancreaticoduodenectomy, *Chemo* chemotherapy, *EPT* ectopic pancreatic tissue, *NR* no recurrence, *LN* lymph node

^a^Reported in years; ^b^Benign tumor such as a polyp

Ectopic pancreatic tissue is found in 0.5–13.7% of laparotomy and autopsy cases and is usually located in the upper gastrointestinal tract [3]. The pathological classification of ectopic pancreas was diagnosed by the Heinrich classification [17]. Malignant transformation of ectopic pancreas tissue most frequently occurs in the upper digestive organs, and the reported incidence ranges from 0.7–1.8% [3]. The following three criteria have been proposed for ectopic pancreas carcinoma: the tumor must be found within or near the ectopic pancreas, a transition between pancreatic structures and carcinoma must be observed, and the non-neoplastic pancreatic tissue must comprise fully developed acini and ductal structures [18]. However, similar to most previous cases, in our case, no ectopic pancreas tissue was identified (Table 1). It has been proposed that a carcinoma arising from ectopic pancreas destroys the primary benign lesion [14].

ACC is typically relatively circumscribed and large with extensive hemorrhage and necrosis [19]. The characteristic microscopic architectural patterns are the acinar pattern, with neoplastic cells arranged in small acinar units, and solid pattern, with solid nests of neoplastic cells lacking luminal formations [20]. In the histopathologic diagnosis of ACC, an immunohistochemical evaluation of pancreatic exocrine enzymes is helpful; both trypsin and chymotrypsin are positive in more than 95% cases [20]. Furthermore, neuroendocrine markers such as chromogranin A, synaptophysin, and CD56 should be assessed when ACC is in the differential diagnosis [9]. In our case, the tumor showed both acinar and solid growth patterns, trypsin-positivity, and neuroendocrine marker-negativity. Therefore, we diagnosed a pure pancreatic-type ACC of the jejunum.

Although ACC has been considered to have a poor prognosis, the surgical resection and 5-year survival rates after resection have been reported as 76.5 and 43.9%, respectively, in a nationwide survey performed in Japan [21]. Furthermore, patients with ACC had a significantly better prognosis than those with pancreatic ductal adenocarcinoma [22]. Aggressive surgical resection with negative margins is also associated with longer survival [23]. However, the prognosis of heterotopic ACC remains unclear because of the limited number of reported cases.

Pancreatic cancers frequently invade the portal venous system leading to extrinsic portal vein obstruction. However, intrinsic venous obstruction by tumor thrombus, while a common occurrence in hepatocellular carcinoma, rarely occurs in pancreatic cancer [16]. Indeed, there have been only a few cases of pancreatic cancer with portal vein tumor thrombus, including two cases of ACC accompanied by tumor thrombus [15, 16]. Furthermore, distinguishing tumor thrombus from a blood clot is very important, because it is well recognized that a tumor thrombus involving the mesenteric venous system is associated with liver metastases. The treatment strategy will also be affected by the preoperative evaluation. In our case, although tumor thrombus was present in the first jejunal vein accompanied by significant mesenteric thickening, we considered the tumor resectable because there were no distant metastases.

The efficacy of neoadjuvant or adjuvant chemotherapy for ACC arising in ectopic pancreatic tissue is still unknown. In patients with unresectable ACC, fluorouracil-based chemotherapy should be considered a neoadjuvant or palliative treatment [24]. However, in the previous 12 reports of heterotopic ACC, only one patient received adjuvant treatment with capecitabine and oxaliplatin after curative resection (Table 1).

In our case, the patient had a high risk of recurrence because of extensive tumor thrombus. Therefore, administered S-1 as adjuvant chemotherapy according to the treatment for pancreatic cancer after curative resection [25]. The patient continued chemotherapy without severe adverse effects or recurrence postoperatively. Future studies are required to assess heterotopic ACC’s sensitivity to chemotherapy and long-term treatment outcomes.

In conclusion, we have presented an extremely rare case of pancreatic-type ACC arising in the jejunum with extensive tumor thrombus into the mesenteric venous system. Although it is difficult to diagnose heterotopic ACC because of its rarity, such lesions may develop in the gastrointestinal tracts as submucosal tumors, sometimes accompanied by tumor thrombus. Curative resection in these cases could be associated with increased survival.

## Abbreviation

ACC: Acinar cell carcinoma
